# Molecular characterisation of *Mycobacterium bovis *isolated from cattle slaughtered at the Bamako abattoir in Mali

**DOI:** 10.1186/1746-6148-4-26

**Published:** 2008-07-17

**Authors:** Borna Müller, Benjamin Steiner, Bassirou Bonfoh, Adama Fané, Noel H Smith, Jakob Zinsstag

**Affiliations:** 1 Swiss Tropical Institute, Basel, Switzerland; 2 Institut du Sahel, Bamako, Mali; 3 Laboratoire Centrale Vétérinaire, Bamako, Mali; 4 Veterinary Laboratories Agency, Weybridge, UK; 5 University of Sussex, Falmer, UK; 6 Centre Suisse de Recherches Scientifiques en Côte d'Ivoire, Abidjan, Côte d'Ivoire

## Abstract

**Background:**

Mali is one of the most important livestock producers of the Sahel region of Africa. A high frequency of bovine tuberculosis (BTB) has been reported but surveillance and control schemes are restricted to abattoir inspections only. The objective of this study was to conduct, for the first time, molecular characterisation of *Mycobacterium bovis *strains isolated from cattle slaughtered at the Bamako abattoir. Of 3330 animals screened only 60 exhibited gross visible lesions. From these animals, twenty strains of *M. bovis *were isolated and characterised by spoligotyping.

**Results:**

Organ lesions typical of BTB were most often detected in the liver, followed by the lung and the peritoneum. *M. bovis *was isolated from 20 animals and 7 different spoligotypes were observed among these 20 strains; three of the patterns had not been previously reported. Spoligotype patterns from thirteen of the strains lacked spacer 30, a characteristic common in strains of *M. bovis *found in Chad, Cameroon and Nigeria. However, unlike the other three Central African countries, the majority of spoligotype patterns observed in Mali also lacked spacer 6. Of the remaining seven strains, six had spoligotype patterns identical to strains commonly isolated in France and Spain.

**Conclusion:**

Two groups of *M. bovis *were detected in cattle slaughtered at the Bamako abattoir. The spoligotype pattern of the first group has similarities to strains previously observed in Chad, Cameroon and Nigeria. The additional absence of spacer 6 in the majority of these strains suggests a Mali specific clone. The spoligotype patterns of the remaining strains suggest that they may have been of European origin.

## Background

Bovine Tuberculosis (BTB) is considered a neglected and poverty related zoonosis [1]. It has a major economic impact on livestock productivity [2], can persist in wildlife reservoirs and thus affect entire ecosystems [3] and it is of public health concern due to its zoonotic potential [4-6]. Although still prevalent in the developed world [7-9], BTB today mostly affects developing countries, which lack the financial and human resources to control the disease [4,5]. The Sahel region of Africa is extremely important in terms of animal production with Mali being amongst the principal cattle producing countries [10,11]. Mali has previously reported a high frequency of BTB but does not apply specific control measures, except carcass inspection at abattoirs [4]. In a recent prevalence study in dairy cattle herds from the peri-urban region of Bamako, 19% of the animals reacted positively to the comparative tuberculin skin test [12].

Spacer oligonucleotide typing (spoligotyping) [13] has been shown to be a valuable tool for investigations of the population structure of *Mycobacterium bovis *in a number of settings [7,8,14-16]. Furthermore, the international designation of spoligotype patterns , [17] has facilitated the comparison of results from different countries and helps elucidate the distribution and spread of strains. Assuming that spoligotype spacers can only be lost and not regained, phylogenetic relationships between strains can be suggested [18,19]. A number of *Mycobacterium tuberculosis *Complex (MTBC) strain families are readily identifiable through spoligotyping [20-22].

Variable number of tandem repeat (VNTR) typing is another simple method for *M. bovis *genotyping with a higher discriminatory power than spoligotyping [19,23]. However, extensive worldwide databases are presently not available and VNTR typing can today mainly be considered a valuable tool for sub-differentiation of strain groups initially identified by spoligotyping [19].

For *M. bovis*, previous studies in Chad, Cameroon and Nigeria have shown that virtually all spoligotype patterns lack spacer 30 with strains bearing spoligotype pattern SB0944 being the most frequent [14-16]. In Cameroon, because of the similarity to patterns of strains isolated in France [7] it was suggested that *M. bovis *could have been imported to this region during the French colonial period [15]. However, strains lacking spacer 30 were so far rarely found outside Chad, Cameroon and Nigeria.

The objectives of this study were to conduct an initial molecular characterisation of *M. bovis *in Mali using spoligotyping and to identify potential exchange of strains with other regions.

## Results

At the abattoir of Bamako, Mali, a case series of 3330 slaughter animals were sequentially screened during standard meat inspection in March and April 2007. A total of 182 specimens from 60 animals with gross visible lesions (apparent lesion prevalence: 1.8%; 95% CI: 1.4 – 2.3%) were collected. Organ lesions were most often detected in the liver (N = 22) followed by the lung (N = 14) and the peritoneum (N = 11). The specimens were put in culture and Acid-Fast Bacilli were further characterised by spoligotyping and typing of the *M. bovis *specific RD4 region. Infection with *M. bovis *was confirmed for 20 animals. From two animals, strains of the *Mycobacterium fortuitum *Complex were isolated as identified by partial sequencing of the 16S rRNA gene. In one case it appeared to be a single infection and in the other case a mixed infection of an *M. fortuitum *Complex strain and *M. bovis*. In this animal, the *M. fortuitum *Complex strain was isolated from liver lesions and *M. bovis *was isolated from lesions of the lungs and bronchial lymph nodes.

**Figure 1:**
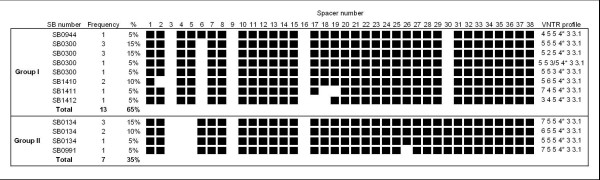
**Spoligotypes and VNTR typing patterns of *M. bovis *strains isolated from slaughter cattle at Bamako abattoir in Mali.** Spacers 39–43 were absent from all spoligotype patterns. SB numbers were taken from . VNTR typing targeted loci ETR A – F [24].

Infection with *M. bovis *was highly associated with the presence of lung lesions (N = 44, chi-squared = 23.7, p < 0.001); in 79% of the animals exhibiting lung lesions, *M. bovis *infection could be confirmed. The association was less strong for liver lesions (N = 48, chi-squared = 3.9, p < 0.05); only 41% of the animals with liver lesions were shown to be infected with *M. bovis*. However, in all 9 cases where *M. bovis *infection was detected in animals with liver lesions, lung lesions were present as well. No association was found between infection with *M. bovis *and lesions in organs other than the liver and lungs. Strains of *M. bovis *isolated in different organs of the same animal showed the same spoligotype pattern. Altogether among the 20 strains of *M. bovis *isolated, seven different spoligotypes were observed; four had been previously reported (SB0944, SB0300, SB0134 and SB0944) and the remaining three were designated SB1410, SB1411 and SB1412 by  (figure 1).

The distinctive lack of spacer 30 was observed in the spoligotype pattern of 13 strains; the majority of those in addition lacked spacer 6 (12/13; figure 1). The remaining seven strains were linked by the absence of spacers 4 and 5 (figure 1).

VNTR typing using the exact tandem repeats (ETR) A-F described by Frothingham et al. [24] allowed to further differentiate strains with the most frequent spoligotype patterns SB0300 and SB0134 (figure 1). Within the strains analysed, only VNTR loci ETR A, B and C showed variation; ETR D, E and F profiles were identical in all the strains. One isolate exhibited two different VNTR alleles (3 and 5 tandem repeats) for locus ETR C (figure 1), indicating either a mixed infection with two distinct strains or a microevolution in this population of strains.

## Discussion

The apparent prevalence of 1.8% gross visible lesions in Malian slaughter cattle was surprisingly low compared to published results from other Sahelian countries [14,25,26] and also lower than the previous tuberculin skin test results of cattle from Bamako indicated (reactor prevalence of 19%) [12]. This suggests that the cattle population slaughtered in the abattoir of Bamako originates largely from extensive pastoral rather than intensive peri-urban production systems, where BTB prevalence is usually higher [27]. Because lesion prevalence was so low and because we could not gauge the sensitivity of lesion detection we did not estimate the true prevalence of BTB at the Bamako abattoir. However, it is likely to be at least two to three fold higher than the observed prevalence [25,26].

Only the presence of lung lesions was strongly associated with the detection of *M. bovis *infection, suggesting that the lung was the primary site of *M. bovis *infection; the association of liver lesions and *M. bovis *infection was weaker. Furthermore, in all the cases where *M. bovis *infection could be confirmed in animals with liver lesions, lung lesions were also present (N = 9). In 12 animals liver lesions were recorded without associated lung lesions, however, in these animals we were unable to isolate *M. bovis *by culture. One animal exhibited a mixed infection of a *M. fortuitum *Complex strain isolated from the liver and *M. bovis*, isolated from lesions associated with the lung and the bronchial lymph nodes. This indicates that some of the lesions outside the lung, and particularly the liver, have been caused by pathogens other than *M. bovis*. The amount of lesion causing infections due to other organisms than *M. bovis *might be considerable as only one third of the animals with lesions could be confirmed to be infected with *M. bovis*. Tuberculous gross visible lesions may be caused by an array of pathogens amongst which Non-tuberculous Mycobacteria (NTM) could play a crucial role in Africa (unpublished results, [28,29]). This suggestion is supported by a previous study in Chad where *M. fortuitum *Complex was repeatedly isolated from lesions in slaughtered cattle [28]. It may be worthwhile to address the role of NTM infections on animal productivity in relation to BTB in African cattle.

Bacteria could only be isolated from 21 out of 60 animals with gross lesions; this was less than in other studies [14,29]. Failure to cultivate bacteria could have been due to long-term storage of tissue samples in the freezer prior to cultivation. Because of frequent power cuts, we cannot exclude that some of the specimen might have undergone multiple freeze-thaw cycles while they were stored. A high amount of completely calcified lesions, without viable tubercle bacilli, could also explain the low recovery of bacteria (references within [26]).

The strains identified here can be divided into two groups by spoligotype pattern (figure 1). The first group is marked by the distinct loss of spacer 30 and accounts for 65% of the strains detected (figure 1). The absence of spacer 30 has previously found to be characteristic of spoligotype patterns for strains of *M. bovis *isolated in Chad, Cameroon and Nigeria [14-16]. In addition to the absence of spacer 30, most of the strains from Mali (12/13) also lack spacer 6, a characteristic not seen in isolates from Central African countries [14-16]. The most often detected *M. bovis *strains in Mali with spoligotype pattern SB0300 could have evolved from strains with spoligotype pattern SB0944 either by drift or a selective sweep. This is supported by the fact that two of the three VNTR types identified in Malian strains with spoligotype pattern SB0300 were identical to VNTR types of previously isolated Nigerian *M. bovis *strains with spoligotype pattern SB0944 [16]. Altogether, the results suggest a close relationship between strains from Mali and those from Central Africa. The spread of related strains over this large area could be explained by the predominant long distance transhumant livestock production system in the Sahel, mainly practised by Fulbe pastoralists [30]. However, considering the fact that, except for pattern SB0944, none of the spoligotype patterns found in Mali are present in any of the three Central African countries and considering that strains with spoligotype pattern SB0944 were rarely detected in Mali we suppose that the spread of *M. bovis *strains over this large distance is relatively slow.

The second group of related spoligotype patterns is characterized by the absence of spacers 4 and 5 (figure 1). The most often detected spoligotype belonging to this group is commonly found in strains from France and Spain (SB0134 [7,31]), suggesting a link between *M. bovis *strains from Mali and mainland Europe. VNTR profile 6 5 5 4 for ETR loci A-D identified in 2/6 Malian strains of *M. bovis *with spoligotype pattern SB0134 has also been detected in a strain isolated from French cattle in the Normandy in 1996 with the same spoligotype pattern [32]. Moreover, three other Malian *M. bovis *strains with ETR A-D profile 7 5 5 4 and spoligotype pattern SB0134 could be closely related to SB0134 *M. bovis *strains with ETR A-D profile 7 4 5 4, which is frequently found in *M. bovis *strains from the Normandy [32]. However, identical spoligotype patterns have also been found in strains from northern Algeria (unpublished results) and livestock migrations from Algeria to Mali through the Sahara desert have been reported. Comprehensive genotyping of strains from West Africa, North Africa and Europe using highly polymorphic markers would be necessary to further elucidate the interrelationship of *M. bovis *strains from these different regions.

Njanpop-Lafourcade et al., have previously suggested an influence of the French colonial history based on the similarity of the predominant spoligotype pattern (SB0944) in Cameroon to the BCG-like spoligotype pattern that is commonly seen in strains from France [15]. In a similar manner it is possible to suggest that strains of *M. bovis *with spoligotype pattern SB0134 were originally imported from Europe. If both assumptions are true this would suggest that either *M. bovis *was not present in the Central or West African region before introduction from Europe to Africa or previously existing "native" *M. bovis *strains have been largely replaced.

Due to the small sample size, the limited survey period and the sampling at only one study location, the *M. bovis *strains collected cannot reflect the country-wide bacterial population structure. Therefore, frequencies of strains with a specific spoligotype may not necessarily mirror the actual frequency of these strains in the population although more frequent strains are also more likely to be detected in a random sample. Moreover, other groups of *M. bovis *strains than the two that were observed may be present in Mali. However, due to the predominant long distance transhumant livestock production system, we believe that the slaughter cattle encountered at the abattoir of Bamako and consequently their associated *M. bovis *strains, represent a sample from a large area of the country.

## Conclusion

This study presents the first molecular characterisation of *M. bovis *strains from Mali. The results suggest that the most often detected strains are related to strains that are prevalent in Chad, Cameroon and Nigeria. A second group of strains shows spoligotype patterns similar to those abundant in mainland Europe and could have been imported directly from Europe or via Northern Africa. Our results can serve as a baseline study for future comparisons with strains from other areas in and around Mali.

## Methods

### Sample collection

Samples were collected from a sequential series of slaughter cattle at the Bamako abattoir, Mali in March and April 2007. The cattle population consisted of crossbreeds between N'Dama, zebu and exotic breeds. The origin of the cattle could not be traced due to poor documentation and multiple selling-on of the animals before slaughter. However, we originally assumed that the cattle originate from the peri-urban region of Bamako as well as the principle areas of cattle production throughout the country. After slaughter, animals underwent a standard meat inspection and organs showing gross visible lesions were confiscated. No ethical clearance was required for this study because it was done on slaughtered animals and organ confiscation was part of a routine monitoring. Tissue samples of 60 animals with gross visible lesions were collected. The samples were transported on ice to the Central Veterinary Laboratory in Bamako. Upon arrival, samples were immediately liberated from connective tissue and fat under a bio-safety cabinet and by use of sterile dissection instruments. The samples were seared on the outside in order to reduce superficial contamination, sealed into sterile stomacher bags and stored at -20°C for maximum one and a half months until they were shipped to Switzerland for culture. Because of frequent power cuts we cannot exclude that some of the specimen might have undergone multiple freeze-thaw cycles while they were stored. Shipment to Switzerland occurred in a refrigerated box; the temperature of the samples was monitored at all times by use of a data-logger (HoboTemp by OnsetCorp) and never exceeded -10°C.

### Tissue preparation, culture and DNA extraction

At the Swiss Reference Centre for Mycobacteria, samples were stored at -80°C until processed. Specimen were dissected and approximately 2 g were homogenised for 2 minutes in 10 ml phosphate buffer saline (PBS) using the ULTRA-TURRAX^® ^Tube Drive homogeniser with DT-20 tubes (IKA, Staufen, Germany). A 5 ml aliquot of the suspension was decontaminated for 15 minutes with 5 ml of decontamination solution (0.5% N-acetyl-L-cystein/2% NaOH/1.45% Na-Citrate solution). The decontamination was stopped by addition of 15 ml PBS, the suspension was centrifuged at 3500 rpm for 15 minutes and the pellet was re-suspended in 2 ml PBS. Then, 0.5 ml of the suspension was added to a BBL™ MGIT™ Mycobacterium Growth Indicator Tube containing OADC enrichment and PANTA™ antibiotic mixture (BD) and incubated in a BACTEC™ MGIT™ 960 Mycobacterial Detection System. In addition, 0.25 ml of the suspension was inoculated onto Löwenstein-Jensen and 7H10 culture media and incubated at 37°C. Cultures were incubated until growth was detected or for at least 8 weeks. Presence of Acid-Fast Bacilli was tested by Ziehl-Neelson staining and Microscopy and DNA of positive cultures was extracted using the InstGene™ Matrix (Bio-Rad^®^).

### Molecular characterisation

Spoligotyping was conducted as previously described [13]. VNTR typing was performed according to the method of Frothingham et al. [24] with adaptations described elsewhere [33]. According to the findings of Brosch et al., strains were confirmed as *M. bovis *by the absence of region RD4. [34]. The 16S rRNA gene amplification and sequencing was carried out as described by Zucol et al., (2006) [35]. Species identification was carried out by comparison with the sequences of the SmartGene Integrated Database Network System (IDNS™) 3.4.0.

### Statistical analysis

Statistical analysis was carried out using Intercooled Stata 9.2 for Windows (StataCorp LP, USA). The association between the presence of lesions in each organ and confirmed *M. bovis *infection was tested by a chi-squared test.

## Authors' contributions

BM: Participated in the conception and design of the study, culture of Mycobacteia, molecular analysis, statistical analysis, writing of the manuscript. BS: Participated in the conception and design of the study, sample collection, culture of Mycobacteria. BB: Participated in the conception and design of the study, acquisition of funds, principle supervision in Mali and intellectual contributions. AF: Principle organisation of sample collection NHS: Contribution in analysis of the data, important intellectual contribution for population genetical analysis. JZ: Principal supervision of the project, principle acquisition of funds, important intellectual contribution for epidemiological questions. All the authors have read and approved the final version of the manuscript.
